# A Family with Von Hippel-Lindau Syndrome: The Findings of Indium-111 Somatostatin Receptor Scintigraphy, Iodine-123 Metaiodobenzylguanidine Scintigraphy and Single Photon Emission Computerized Tomography

**DOI:** 10.4274/mirt.70894

**Published:** 2017-02-01

**Authors:** Pelin Arıcan, Berna Okudan Tekin, Seniha Naldöken, Rıza Şefizade, Dilek Berker

**Affiliations:** 1 Ankara Numune Training and Research Hospital, Clinic of Nuclear Medicine, Ankara, Turkey; 2 Ankara Numune Training and Research Hospital, Clinic of Endocrinology and Metabolism, Ankara, Turkey

**Keywords:** Von Hippel-Lindau disease, neuroendocrine tumors, scintigraphy, single-photon emission computerized tomography

## Abstract

Von Hippel-Lindau syndrome (VHLS) is an autosomal dominant hereditary familial disorder characterized by development of malignant and benign neoplasms. Differential diagnosis of the adrenal and pancreatic masses are difficult in patients with VHLS. Iodine-123 metaiodobenzylguanidine (I-123 MIBG) and indium-111 somatostatin receptor scintigraphies (In-111 SRS) have important roles in the differential diagnosis of adrenal and pancreatic masses in those patients. In this case report, we present the findings of I-123 MIBG single-photon emission computerized tomography (SPECT/CT) and In-111 SRS SPECT/CT in three members of a family with VHLS. In case 1, a residual neuroendocrine tumor (NET) was detected in the head of pancreas on In-111 SRS SPECT/CT images. In case 2 and 3, I-123 MIBG SPECT/CT confirmed the adrenal masses as pheochromocytoma, and the extra-adrenal mass as NET, before surgery. We thought that In-111 SRS and I-123 MIBG scan might be helpful in the routine work up of VHLS patients for diagnostic and therapeutic purposes. Hybrid SPECT/CT system may improve diagnostic accuracy of planar images since it assesses morphologic and functional information together.

## Abstract (tr)

Von Hippel-Lindau sendromu (VHLS) malign ve benign tümörler ile karakterize otozomal dominant herediter ailesel bir hastalıktır. VHLS’li hastalarda adrenal ve pankreatik kitlelerin ayırıcı tanısı zordur. Bu hastalarda iyot-123 metaiyodobenzilguanidin (I-123 MIBG) ve indiyum-111 somatostatin reseptör sintigrafileri (In-111 SRS) adrenal ve pankreatik kitlelerin ayırıcı tanısında önemli rol oynar. Bu olgu sunumunda VHLS’li ailenin üç üyesinin I-123 MIBG bilgisayarlı tek foton emisyonlu tomografisi (SPECT/BT) ve In-111 SRS SPECT/BT bulgularını sunuyoruz. Olgu 1’de, In-111 SRS SPECT/BT görüntülerinde pankreas başında rezidü nöroendokrin tümör (NET) saptandı. Olgu 2 ve 3’de, I-123 MIBG SPECT/BT cerrahi öncesi adrenal bezdeki kitleleri feokromositoma ve adrenal dışı kitleyi NET olarak onayladı. In-111 SRS ve I-123 MIBG sintigrafilerinin VHLS’li hastaların tanı ve tedavisi için rutin kullanımda yararlı olabileceğini düşündük. Hibrid SPECT/BT morfolojik ve fonksiyonel bilgileri birlikte değerlendirdiği için, planar görüntülerin tanısal doğruluğunu artırır.

## INTRODUCTION

Von Hippel-Lindau syndrome (VHLS) is an autosomal dominant hereditary familial neoplastic disorder characterized by retinal, cerebellar and spinal hemangioblastomas, liver, and kidney hemangiomas, clear cell renal carcinoma (RCC), pheochromocytoma, pancreatic tumors, pancreatic, renal, and epididymal cysts (1). The gene responsible for the disease is identified with direct gene mutation analysis (2). Clinic examination and imaging modalities are important in the diagnosis of VHLS (3). Nuclear medicine imaging modalities such as iodine-123 metaiodobenzylguanidine (I-123 MIBG) and Indium-111 somatostatin receptor scintigraphy (In-111 SRS) have important roles in the differential diagnosis of adrenal and pancreatic masses in those patients.

Here in, we present the findings of I-123 MIBG single photon emission computerized tomography (SPECT/CT) and In-111 SRS SPECT/CT in three members of a family with VHLS.

## CASE REPORTS

### Case 1

**Figure 1:**
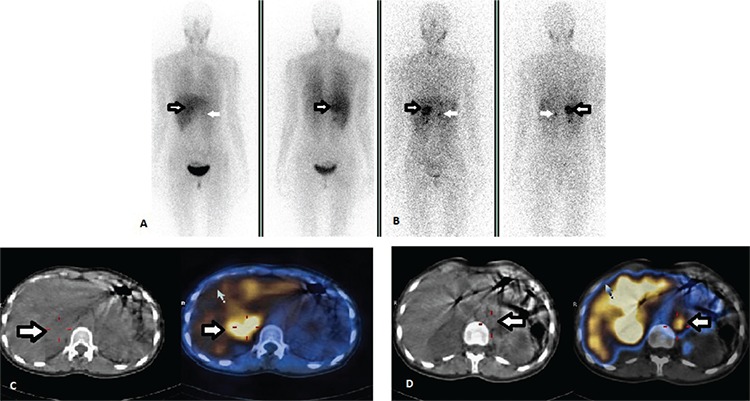
In-111 somatostatin receptor scintigraphies findings of case 1. Anterior-posterior whole body images show focal radiotracer accumulation medial to the right kidney (A) at 4, and (B) 24 hours (arrows). Axial (C) and coronal (D) computed tomography (CT), single photon emission computerized tomography (SPECT), and SPECT/CT images show that this accumulation is localized to the head of the pancreas (arrows). The diagnosis of residual neuroendocrine tumor was confirmed by histopathologic examination

A 39-year-old man with VHLS who had had adrenalectomy for pheochromocytoma 27 years ago, partial pancreatectomy for neuroendocrine tumor (NET) 6 years ago, and partial nephrectomy for RCC 5 years ago was referred to our department for evaluation of a residual mass in his pancreas. The contrast-enhanced abdominal CT revealed a 30-mm mass at the head of the pancreas. In-111 SRS imaging was performed after injection of 185 MBq In-111 octreotide (Octreoscan, Mallinckrodt, Netherlands). Whole body and static images were obtained 4 and 24 hours later. Focal radiotracer accumulation was seen in the right upper quadrant of the abdomen on planar images. Therefore, abdominal SPECT/CT was performed following planar imaging to the abdominal region (Millennium Hawkeye 4, GE Medical Systems, Wilwaukee, WI). SPECT/CT confined this pathologic uptake to the pancreatic region (Figure 1). The patient had revision surgery. Histolopathologic examination revealed a recurrent NET.

### Case 2

**Figure 2:**
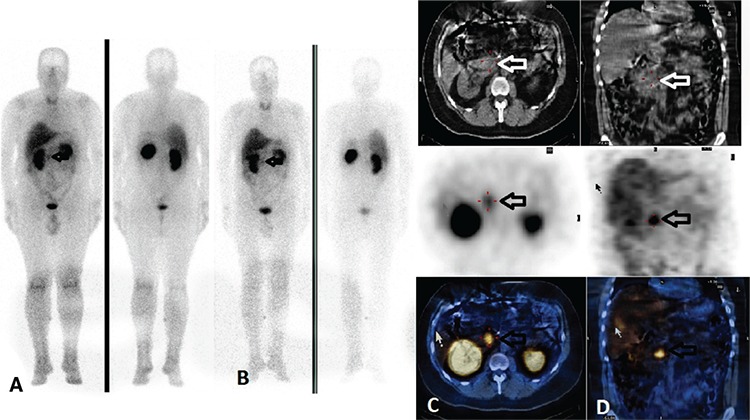
Iodine-123 metaiyodobenzilguanidin scintigraphy findings of case 2. Anterior-posterior whole body images show focal tracer uptake superior to the kidneys bilaterally (A) at 4, and (B) 24 hours, before surgery (arrows), (C) axial (D) coronal computed tomography (CT), single photon emission computerized tomography (SPECT), and SPECT/CT images reveal that those radiotracer accumulations are localized to the adrenal glands bilaterally (arrows). Histopathologic examination of the masses were reported as pheochromocytoma after surgery

The cousin of case 1, a 20-year-old woman was diagnosed with VHLS on gene mutation analysis. Hypertension and increased metanephrine levels were detected on psychical examination and laboratory tests. Magnetic resonance imaging (MRI) detected a hemangioma in the liver and masses in bilateral adrenal glands (right side: 28x22 mm, and left side: 20x18 mm). I-123 MIBG scintigraphy was performed before surgery to confirm adrenal masses as pheochromocytoma. Whole body and static images were obtained 4 and 24 hours after injection of 370 MBq I-123 MIBG (I-123, Mallinckrodt, Netherlands). Planar images showed an abnormal radiotracer uptake bilaterally superior to kidneys. SPECT/CT fusion images demonstrated that those radiotracer accumulations were localized to the adrenal glands (Figure 2). Bilateral adrenalectomy was performed. Histopathologic examination revealed that the removed adrenal tumors were pheochromocytoma. Four years later, abdominal MRI revealed a mass between the liver and right adrenal gland (33x30x36 mm) and another one in the pancreatic tail (13x15x17 mm). I-123 MIBG SPECT/CT was performed with suspicion of local recurrence. Fusion images showed an abnormal accumulation in the right adrenal gland. There was no radiotracer accumulation in the pancreas, a Ga-68 DOTATOC positron emission tomography (PET/CT) was performed. Pathologic radiotracer uptake was demonstrated in the pancreas. The patient is planned to undergo surgery.

### Case 3

**Figure 3:**
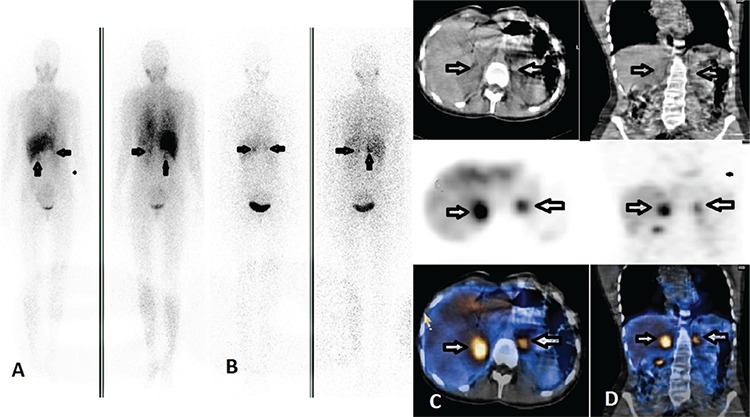
Iodine-123 metaiyodobenzilguanidin scintigraphy findings of case 3. Anterior-posterior whole body images show a large focal tracer uptake medial to the liver (arrows with black contours) and small focal activity superior to the left kidney (white arrows) (A) at 4, and (B) 24 hours, before surgery. Axial computed tomography (C), and single photon emission computerized tomography slices show the intense abnormal radiotracer accumulation in the right adrenal gland (arrows), and (D) a smaller activity in the extra adrenal and prevertebral region (arrows). After surgery, the histopathologic examination confirmed the mass in the right adrenal as pheochromocytoma, and the mass on the left side as paraganglioma

Case 3 was a 38-year-old woman who is the cousin of both case 1 and 2. She had no complaints, and her parameters were in normal limits. She was diagnosed with VHLS on gene mutation analysis. An abdominal MRI was performed for screening purposes. MRI revealed masses with intensive contrast enhancement between the liver and right kidney (40x36x36 mm), and inferior to the left adrenal gland (14x15x15 mm). Those findings were suspicious for bilateral pheochromocytoma. I-123 MIBG was performed to characterize those tumors. Whole body and static images were obtained 4 and 24 hours after injection of 370 MBq I-123 MIBG. Whole body-scan and static planar images showed an abnormal radiotracer uptake infero-medial to the liver, and supero-medial to the left kidney. Following planar imaging, an abdominal SPECT/CT was performed. SPECT/CT images showed radiotracer uptake in the right adrenal gland region. The activity on the left side was localized to the extra-adrenal region (Figure 3). Right adrenalectomy and resection of the extra-adrenal lesion were performed following MIBG scan. Histopathologic examination confirmed the diagnosis of pheochromocytoma in the right adrenal gland, and paraganglioma in the extra-adrenal tumor.

Three members of this family with VHLS are still being followed up in the endocrinology department.

## LITERATURE REVIEW AND DISCUSSION

VHLS is an autosomal dominant disorder characterized by presence of malignant and benign neoplasms. Clinical diagnosis of VHLS is usually challenging due to involvement of various organs. Early detection of neoplasms is important to reduce morbidity. Adrenal glands and pancreas are usually involved in those patients. Clinical and laboratory findings facilitate the diagnosis. CT and MRI can localize adrenal, extra-adrenal, and pancreatic tumors. However, those imaging modalities can not characterize the functional status of the tumors. In-111 SRS is very useful for both the diagnosis and staging of pancreatic NET, and management of patients (4). Pancreatic tumors are detected in 5-10% of the cases with VHLS. In case 1, In-111 SRS SPECT/CT fusion images confirmed residual NET in the head of pancreas, which was previously detected with abdominal CT. The findings assisted to plan the treatment. The patient had a revision surgery. He was re-operated. Pulcrano et al. (5) reported the findings of In-111-DTPA0-octreotide scintigraphy in three members of a family with VHLS. The authors concluded that scintigraphy might be useful in the management plan of those patients. Recently, PET/CT performed using Ga-68 labelled peptides has been proven to be very useful for imaging of NET. Sharma et al. (3) reported the findings of Ga-68 DOTA-NOC PET/CT in a patient with VHLS. They demonstrated uptake of Ga-68 DOTA-NOC in all central nervous system and visceral tumors. In addition, Ga-68 DOTA-NOC PET/CT assisted the decision of treating the patient with Lu-177 DOTA-TATE. The authors suggested that Ga-68 DOTA-NOC PET/CT played an important role in the management and diagnosis of patients with VHLS, and that it should be used routinely in those patients.

I-123 MIBG scintigraphy is the imaging modality of choice in pheochromocytoma owing to its high sensitivity and specificity. I-123 MIBG is performed prior to surgery both to confirm the lesions as pheochromocytoma, and to determine metastatic, residual or recurrent disease (4). In case 2, I-123 MIBG SPECT/CT confirmed bilateral adrenal masses as pheochromocytoma before bilateral adrenalectomy was performed. Four years after surgery, I-123 MIBG SPECT/CT showed local recurrence in bilateral adrenal glands. Fujita et al. (6) reported a similar case with VHLS. Recurrence of bilateral pheochromocytoma was determined with I-123 MIBG scan in that case. Thoren et al. (7) reported a case with multiple pheochromocytomas and VHLS. They performed I-123 MIBG before surgery to confirm that the lesions were indeed pheochromocytoma and to rule out any metastatic disease. Arao et al. (8) confirmed the diagnosis of bilateral pheochromocytoma with MIBG scintigraphy in a case with VHLS. In our report, case 3 had no symptoms or history of hypertension. Her laboratory parameters were within normal limits. Abdominal MRI revealed bilateral adrenal masses with significant I-123 MIBG accumulation. Fusion images confirmed the diagnosis of pheochromocytoma before surgery. I-123 MIBG SPECT/CT was helpful in the early diagnosis of this asymptomatic case. Otsuka et al. (9) described a similar case with VHLS who did not have any symptoms and had significant accumulation of I-131 MIBG in bilateral pheochromocytoma.

In conclusion, the data we obtained from our patients suggested that In-111 SRS and I-123 MIBG scan might be helpful in the routine work up of VHLS patients for diagnostic and therapeutic purposes. Hybrid SPECT/CT system can improve the diagnostic accuracy of In-111 SRS and I-123 MIBG images, since it evaluates morphologic and functional information together. SPECT/CT increases sensitivity and specificity of planar images.
